# Cumulative incidence and prognostic factors for leukemic transformation in chronic myelomonocytic leukemia: a competing risk analysis

**DOI:** 10.1186/s12885-026-16346-y

**Published:** 2026-06-17

**Authors:** Jungao Huang, Chaoqiang Zheng, Yulan Liu, Changhao Li, Xiaoqin Xin

**Affiliations:** 1 Department of Medical Genetic, Ganzhou Maternal and Child Health Hospital, Ganzhou, Jiangxi Province 341000 China; 2 Department of Clinical Laboratory, Ganzhou People’s Hospital, Ganzhou, Jiangxi Province China

**Keywords:** Chronic myelomonocytic leukemia, Competing risk, Leukemic transformation, Fine-Gray model, Cumulative incidence, Hypomethylating agents, Allogeneic transplantation

## Abstract

**Background:**

Leukemic transformation represents a pivotal event in the natural history of chronic myelomonocytic leukemia (CMML), yet conventional survival analysis may yield biased estimates by treating non-transformation deaths as censored observations. We hypothesised that competing risk methodology would provide more accurate transformation estimates and could alter the interpretation of treatment-associated effects compared with traditional approaches.

**Methods:**

We retrospectively analysed 102 patients with CMML diagnosed and treated at Ganzhou People’s Hospital between January 2014 and November 2025. Cumulative incidence functions (CIF) were estimated using the Aalen–Johansen method with death without transformation as the competing event. Fine–Gray subdistribution hazard models identified independent predictors, with parallel Cox regression for methodological comparison. Treatments were classified by primary modality at baseline (HMA, chemotherapy, best supportive care (BSC), allo-HSCT) and entered as a stratification covariate rather than as a randomised exposure.

**Results:**

During follow-up, 54 patients (52.9%) developed acute myeloid leukemia and 7 (6.9%) died without transformation. The 12-, 24-, and 36-month CIF were 46.9%, 63.4%, and 73.8%, respectively, whereas 1 − Kaplan–Meier estimates were 49.2%, 67.2%, and 78.4% (4.9–6.2% relative overestimation). Baseline treatment strategy was associated with transformation risk: compared with HMA, BSC showed a higher subdistribution hazard (SHR 4.77, 95% CI 1.86–12.22, *P* = 0.001) and chemotherapy SHR 2.16 (1.04–4.48, *P* = 0.039), with allo-HSCT not significantly associated (SHR 0.52, *P* = 0.261); these associations should be interpreted as exploratory given non-random allocation and small subgroup sizes (allo-HSCT *n* = 13, BSC *n* = 22). Intermediate and high-risk karyotypes independently predicted transformation (SHR 3.09 and 2.98, both *P* < 0.05). Within the NGS-tested subgroup, *SETBP1* (SHR 3.09, *P* = 0.009) and *TP53* (SHR 3.66, *P* = 0.009) mutations marked small subsets at very high transformation risk. For allo-HSCT, Cox regression yielded a significant protective effect (HR 0.31, *P* = 0.026) that became non-significant in the Fine–Gray model, illustrating how cause-specific analysis can overstate the isolated anti-transformation effect of a treatment that simultaneously reduces mortality.

**Conclusions:**

This study shows how competing-risk analysis complements standard Kaplan–Meier estimates of overall and progression-free survival when the cumulative incidence of a specific event, leukaemic transformation, is the quantity of interest. Although the modest, single-centre sample and non-randomised treatment allocation preclude definitive treatment recommendations, cytogenetic risk was the strongest disease-related prognostic factor, and molecular profiling provided additional risk discrimination within the NGS-tested subgroup. Cumulative incidence functions should therefore be reported routinely alongside Kaplan–Meier estimates in CMML and other myeloid neoplasms with non-negligible competing mortality.

**Supplementary Information:**

The online version contains supplementary material available at 10.1186/s12885-026-16346-y.

## Introduction

Chronic myelomonocytic leukemia (CMML) is a clonal stem-cell disorder with overlapping myelodysplastic and myeloproliferative features. Leukemic transformation to acute myeloid leukemia occurs in 14–30% of patients and carries a dismal prognosis, with median survival measured in months [1–3]; sequential acquisition of mutations in *ASXL1*, *RUNX1*, *NRAS* and related genes has been implicated in this progression [4, 5]. First-line treatment include hypomethylating agents (HMAs), intensive chemotherapy, allogeneic haematopoietic stem-cell transplantation (allo-HSCT) and best supportive care (BSC), with selection driven by age, fitness and donor availability [6–9]. Accurate estimation of transformation risk is therefore central to the timing and intensity of disease-modifying therapy, particularly the threshold for transplantation.

Most published transformation estimates in CMML are derived from Kaplan–Meier (KM) analysis or cause-specific Cox regression, both of which censor patients who die without transformation [10, 11]. This is valid only if censored patients would have transformed at the same rate as those still under observation—an assumption violated when death precludes subsequent transformation. The resulting overestimation accumulates over time and is most pronounced in older populations with substantial competing mortality [12–14].

The competing risk framework provides an unbiased alternative. Cumulative incidence functions (CIFs) estimated by the Aalen–Johansen method give marginal event probabilities that respect competing outcomes [15, 16], and the Fine–Gray subdistribution hazard model extends this approach to multivariable regression [17]. Although routinely applied in transplantation, cardiovascular disease and solid-tumour oncology, competing risk methodology remains underused in myeloid malignancies [16, 18], and its effect on transformation estimates in CMML has not been systematically evaluated.

We applied competing risk methodology to a single-centre CMML cohort to (1) estimate the cumulative incidence of leukemic transformation accounting for competing mortality; (2) quantify the bias introduced by KM analysis; (3) identify independent predictors using Fine–Gray regression and compare them with cause-specific Cox estimates; and (4) describe transformation risk across baseline treatment strategies and disease characteristics, with an exploratory analysis of recurrent mutations in the NGS-tested subgroup.

## Methods

### Study design and patients

**Fig. 1:**
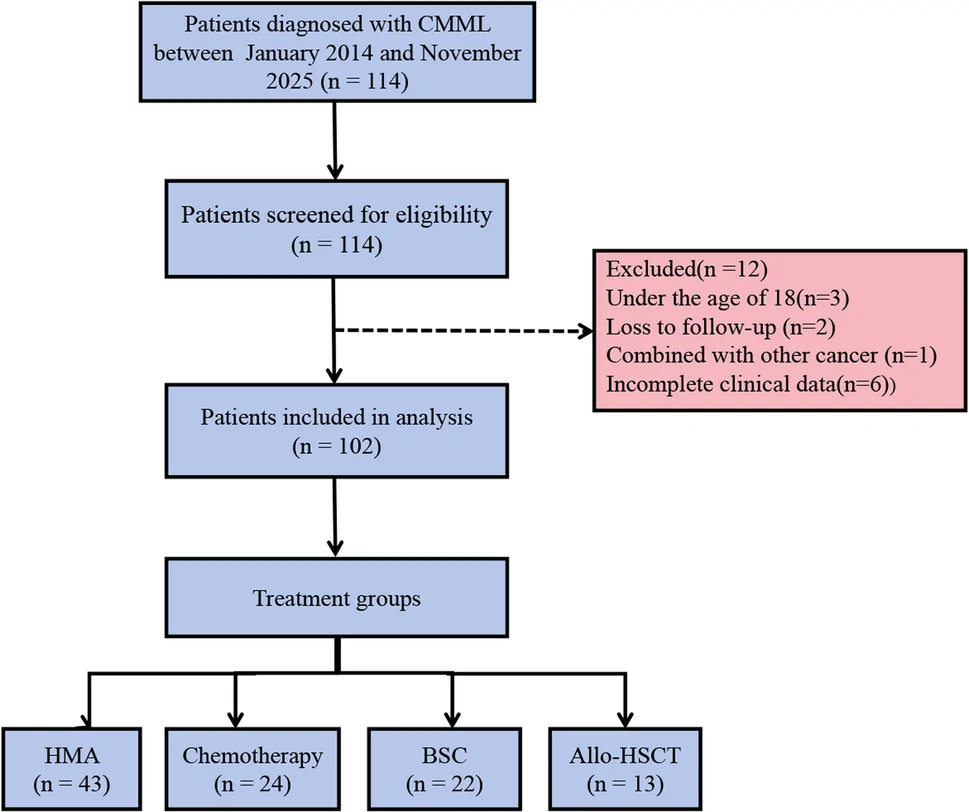
Study flow diagram. A total of 114 patients diagnosed with CMML between January 2014 and November 2025 were screened. Twelve patients were excluded due to age < 18 years (*n* = 3), early loss to follow-up (*n* = 2), concurrent other cancer (*n* = 1), or incomplete data (*n* = 6). The final cohort of 102 patients was divided into four treatment groups: hypomethylating agents (HMA, *n* = 43), chemotherapy (*n* = 24), best supportive care (BSC, *n* = 22), and allogeneic stem cell transplantation (Allo-HSCT, *n* = 13)

We conducted a retrospective cohort study at Ganzhou People’s Hospital. Consecutive adults (≥ 18 years) diagnosed with chronic myelomonocytic leukemia (CMML) according to the fifth-edition WHO classification [19] between January 2014 and November 2025 were identified by systematic review of the institutional database. Patients with prior therapy for a myeloid malignancy, a concurrent solid tumor requiring active treatment, or follow-up < 1 month were excluded. Of 114 patients screened, 12 were excluded (age < 18, *n* = 3; follow-up < 1 month, *n* = 2; concurrent malignancy, *n* = 1; incomplete clinical data, *n* = 6), leaving 102 patients for analysis (Fig. 1).

Because CMML is a rare neoplasm, no formal a priori sample-size calculation was performed; all consecutive eligible patients within the pre-specified 11.9-year enrolment window were included. The study was approved by the Ganzhou People’s Hospital Ethics Committee (PJB2026-016-01) and conducted in accordance with the Declaration of Helsinki; the requirement for informed consent was waived given the retrospective design.

### Baseline assessment

Baseline workup comprised complete blood count with differential, bone marrow aspirate and biopsy with conventional cytogenetics, and serum chemistry. Fluorescence in situ hybridization supplemented karyotyping when metaphases were insufficient. Targeted next-generation sequencing (NGS) for myeloid driver mutations ( including *ASXL1*, *RUNX1*, *NRAS*, *SETBP1*, *SRSF2*, *TET2* and *TP53*) was performed in 62 patients (60.8%) when clinically indicated and reimbursable; mutations were detected in 60 of these (96.8%), and the molecular analyses are therefore restricted to this NGS-tested subgroup rather than incorporated into the primary multivariable model.

### Disease classification and risk stratification

Patients were classified by the fifth-edition WHO criteria (CMML-1, CMML-2) and the FAB system (MD-CMML, MP-CMML) [20]. Cytogenetic risk followed the CMML-specific Prognostic Scoring System (CPSS) [21]: low risk (normal karyotype, isolated − Y); high risk (trisomy 8, any chromosome 7 abnormality, complex karyotype defined as ≥ 3 abnormalities); intermediate risk (all other clonal abnormalities).

### Treatment groups

Primary treatment was assigned by multidisciplinary review on the basis of disease characteristics, performance status, comorbidity burden and patient preference, and was used for analytic stratification:

Cytotoxic chemotherapy (*n* = 24): cytarabine-based induction (7 + 3 or equivalent) given during the CMML phase before any morphologic transformation.

Hypomethylating agents (HMA; *n* = 43): azacitidine 75 mg/m² subcutaneously on days 1–7, or decitabine 20 mg/m² intravenously on days 1–5, of each 28-day cycle, continued until progression or unacceptable toxicity. The reference category for treatment was set as HMA because HMA was the most frequently used disease-modifying therapy in the cohort (*n* = 43) and provided the largest, most stable reference stratum.

Best supportive care (BSC; *n* = 22): transfusion support, erythropoiesis-stimulating agents, hydroxyurea for proliferative symptoms and antimicrobial prophylaxis; this group comprised both patients unsuitable for or declining disease-modifying therapy and those with asymptomatic lower-risk CMML-1 managed expectantly.

Allogeneic hematopoietic stem-cell transplantation (allo-HSCT; *n* = 13): myeloablative or reduced-intensity conditioning per institutional protocols. Patients who received induction chemotherapy and subsequently proceeded to transplant were classified in the allo-HSCT group.

### Endpoints

The primary endpoint was time to leukemic transformation, defined as progression to AML with ≥ 20% blasts in bone marrow or peripheral blood per WHO criteria [19] and confirmed morphologically in all cases. Death without prior transformation was treated as the competing event. Patients alive and transformation-free were censored at last follow-up. Time was measured from the date of CMML diagnosis. Overall survival, measured from diagnosis to death from any cause, was a secondary endpoint.

### Statistical analysis

Continuous variables are reported as medians (IQR) and categorical variables as counts (%); between-group comparisons used Kruskal–Wallis tests and chi-square or Fisher exact tests, as appropriate.

Cumulative incidence functions (CIF) for leukemic transformation were estimated with the Aalen–Johansen estimator, treating death without transformation as a competing event [15]. For methodological comparison, 1 − Kaplan–Meier (1 − KM) estimates were also computed; the difference between the two estimators quantified the bias introduced by ignoring competing risks. Group differences in CIF were tested with Gray’s test.

Fine–Gray proportional subdistribution hazards regression estimated subdistribution hazard ratios (SHR) for transformation in the presence of competing mortality [16]; cause-specific hazard ratios (HR) from Cox regression were reported in parallel. The multivariable model used a two-step strategy: WHO classification, karyotype risk and treatment arm (HMA as reference, selected as the largest disease-modifying stratum) were forced in a priori, regardless of significance; remaining laboratory and clinical covariates with *P* < 0.10 on univariate analysis underwent backward elimination at *P* < 0.10, retaining bone marrow blast percentage and white blood cell count in the final model.

Proportional hazards were assessed by scaled Schoenfeld residuals for the Cox model and by time-by-covariate interactions for the Fine–Gray model following Austin and Fine [17]; no violations were detected, and time-fixed coefficients were retained (Supplementary Table S1). Linearity of continuous covariates was confirmed with restricted cubic splines. Analyses were performed in R 4.3.0 (*cmprsk* and *survival* packages). Two-sided *P* < 0.05 was considered statistically significant.

## Results

### Patient characteristics

The cohort comprised 102 CMML patients diagnosed between January 2014 and November 2025 (Fig. 1; Table 1). Median age was 64.5 years (IQR 56.0–71.8) and 27 patients (26.5%) were female. By WHO criteria, 53 (52.0%) had CMML-1 and 49 (48.0%) CMML-2; by FAB, 42 (41.2%) MD-CMML and 60 (58.8%) MP-CMML. Karyotype was low-risk in 74 (72.5%), intermediate in 13 (12.7%), and high-risk in 15 (14.7%). First-line treatment was HMA in 43 (42.2%), chemotherapy in 24 (23.5%), BSC in 22 (21.6%), and allo-HSCT in 13 (12.7%). Allo-HSCT recipients were younger (median 56.0 vs. 65.0–69.0 years, *P* = 0.011) and BSC patients had lower albumin (32.4 g/L, *P* < 0.001); WHO, FAB, and karyotype distributions showed no statistically significant differences across treatment groups (all *P* > 0.08; Table 1).

**Table 1:** Baseline characteristics by treatment group

Variable	Chemotherapy (*n* = 24)	HMAs (*n* = 43)	BSC (*n* = 22)	Allo-HSCT (*n* = 13)	Total (*n* = 102)	*P*
Age, years	65.5 (58.0–71.0)	65.0 (56.5–71.5)	69.0 (59.2–77.0)	56.0 (52.0–59.0)	64.5 (56.0-71.8)	0.011
Gender						0.605
Female	7 (29.2)	11 (25.6)	4 (18.2)	5 (38.5)	27 (26.5)	
Male, *n* (%)	17(71.8)	32(74.4)	18(81.8)	8(61.5)	75(73.5)	
WHO classification						0.482
CMML-1	11 (45.8)	24 (55.8)	13 (59.1)	5 (38.5)	53 (52.0)	
CMML-2	13 (54.2)	19 (44.2)	9 (40.9)	8 (61.5)	49 (48.0)	
FAB classification						0.194
MD-CMML	12 (50.0)	13 (30.2)	10 (45.5)	7 (53.8)	42 (41.2)	
MP-CMML	12 (50.0)	30 (69.8)	12 (54.5)	6 (46.2)	60 (58.8)	
Karyotype risk						0.083
Low	19 (79.2)	30 (69.8)	15 (68.2)	10 (76.9)	74 (72.5)	
Intermediate	3 (12.5)	2 (4.7)	6 (27.3)	2 (15.4)	13 (12.7)	
High	2 (8.3)	11 (25.6)	1 (4.5)	1 (7.7)	15 (14.7)	
BM blast, %	11.0 (6.0-14.2)	9.5 (5.0-13.7)	7.5 (3.3–12.3)	11.0 (4.1–14.0)	10.0 (4.1–13.5)	0.399
WBC, ×10⁹/L	19.9 (6.6–41.0)	19.6 (12.5–37.4)	19.0 (6.4–38.0)	11.3 (6.2–22.1)	17.4 (8.0-37.6)	0.482
Hemoglobin, g/L	82.0 (71.8-112.2)	85.0 (69.0-103.5)	76.0 (58.2–83.5)	101.0 (71.0-120.0)	81.5 (67.0-108.0)	0.060
Platelet, ×10⁹/L	84.0 (26.5–159.0)	94.0 (39.5–166.0)	59.0 (38.2-125.5)	69.0 (52.0-177.0)	74.0 (38.2–171.0)	0.819
LDH, U/L	282.5 (221.7-508.9)	302.4 (214.2-487.2)	275.0 (230.0-448.0)	213.5 (136.5-335.1)	280.0 (202.6–448.0)	0.254
Albumin, g/L	39.1 (36.6–44.9)	38.7 (33.5–41.6)	32.4 (29.7–39.3)	44.2 (38.1–45.3)	38.6 (33.2–42.7)	< 0.001
AML transformation	19 (79.2)	21 (48.8)	10 (45.5)	4 (30.8)	54 (52.9)	0.013
Death	13 (54.2)	14 (32.6)	9 (40.9)	5 (38.5)	41 (40.2)	0.388
Follow-up time, month	14 (9–21)	12 (9–20)	5(4–7.5)	24(18–29)	12(6–20)	< 0.001

Continuous variables: median (IQR); Categorical variables: *n* (%)

*HMAs* Hypomethylating agents, *HSCT* Allogeneic hematopoietic stem cell transplantation, *BSC* Best supportive care, *CMML* Chronic myelomonocytic leukemia, *FAB* French- American- British, *WHO* World Health Organization, *BM* Bone marrow, *WBC* White blood cell count, *LDH* Lactate dehydrogenase

### Events and follow-up

Median follow-up was 12.0 months (IQR 6.0–20.0), longest for allo-HSCT (24.0) and shortest for BSC (5.0). Leukemic transformation occurred in 54 patients (52.9%); 7 (6.9%) died without transformation (competing event); 41 (40.2%) remained transformation-free. Crude transformation differed by treatment (*P* = 0.013): chemotherapy 79.2%, HMA 48.8%, BSC 45.5%, allo-HSCT 30.8%.

### Univariate predictors

Univariate Fine–Gray estimates are shown in Table 2. WHO CMML-2 (SHR 1.79, 95% CI 1.04–3.08, *P* = 0.036), bone-marrow blast percentage (SHR 1.10 per 1%, 1.04–1.16, *P* = 0.001), WBC (SHR 1.06 per 10 × 10⁹/L, 1.01–1.11, *P* = 0.022), LDH (SHR 1.08 per 100 U/L, 1.00–1.16, *P* = 0.041), and lower hemoglobin (SHR 0.88 per 10 g/L, 0.79–0.97, *P* = 0.014) predicted transformation. Relative to HMA, BSC conferred a significantly higher subdistribution hazard (SHR 2.80, 1.24–6.31, *P* = 0.013), whereas chemotherapy (SHR 1.80, 0.96–3.37, *P* = 0.067) and allo-HSCT (SHR 0.45, 0.15–1.31, *P* = 0.143) did not reach significance; high-risk karyotype showed a similarly non-significant association (SHR 1.87, 0.96–3.61, *P* = 0.064). FAB classification, age, sex, platelets, and albumin were not associated (all *P* > 0.10). The proportional subdistribution hazards assumption held for all retained covariates (global χ²=3.42, df = 8, *P* = 0.91; Supplementary Table S1).

**Table 2:** Univariate Fine–Gray analysis for AML transformation

Variable	SHR	95% CI	*P* value
Gender (Male vs. Female)	1.16	0.66–2.05	0.605
WHO (CMML-2 vs. CMML-1)	1.79	1.04–3.08	0.036
FAB (MP vs. MD)	1.30	0.75–2.23	0.351
Karyotype risk (vs. Low)			
Intermediate	1.46	0.65–3.31	0.360
High	1.87	0.96–3.61	0.064
Treatment (vs. HMA)			
Chemotherapy	1.80	0.96–3.37	0.067
BSC	2.80	1.24–6.31	0.013
Allo-HSCT	0.45	0.15–1.31	0.143
Age (per year)	0.99	0.97–1.01	0.432
BM blast (per %)	1.10	1.04–1.16	0.001
WBC (per 10 × 10⁹/L)	1.06	1.01–1.11	0.022
Hemoglobin (per 10 g/L)	0.88	0.79–0.97	0.014
Platelet (per 50 × 10⁹/L)	1.02	0.91–1.15	0.679
LDH (per 100 U/L)	1.08	1.00-1.16	0.041
Albumin (per g/L)	0.97	0.92–1.01	0.133

*SHR* Subdistribution Hazard Ratio, *CI* Confidence Interval

### Multivariate predictors

**Fig. 2:**
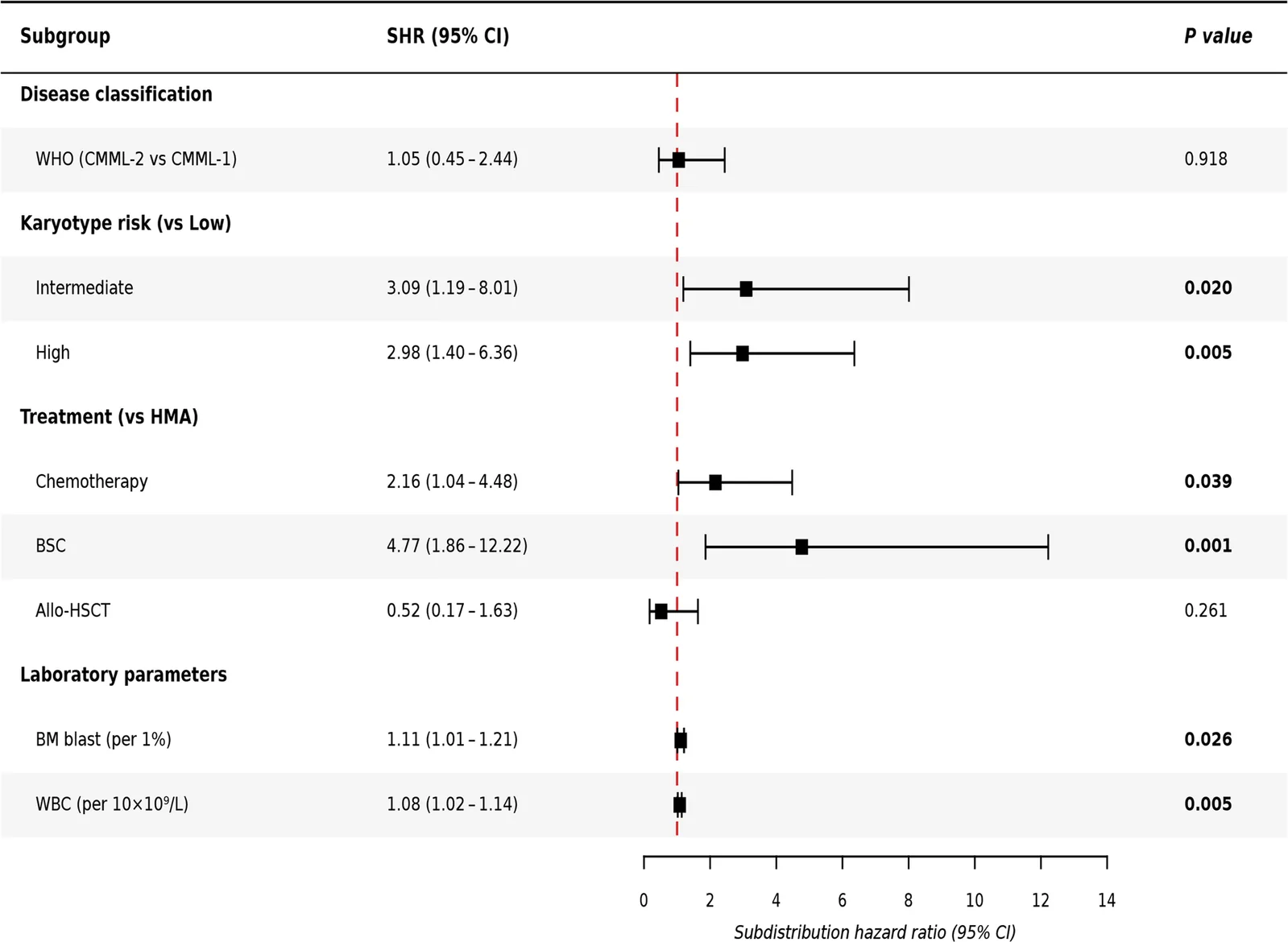
Multivariable Fine–Gray subdistribution hazards model for leukemic transformation in chronic myelomonocytic leukemia. Forest plot of subdistribution hazard ratios (SHRs) with 95% confidence intervals and corresponding *P* values from the multivariable Fine–Gray model (*n* = 102; 54 transformation events, 7 competing deaths), with death without prior transformation treated as the competing event. Reference categories were CMML-1 for WHO classification, low-risk karyotype, and hypomethylating agents (HMA) for treatment. Bone-marrow blast percentage and white blood cell count were modelled as continuous variables, with SHRs expressed per 1% absolute increase and per 10×10⁹/L increase, respectively. Squares denote point estimates and horizontal lines represent 95% confidence intervals; the vertical reference line at SHR = 1 indicates no association. Estimates with confidence intervals excluding 1 (intermediate- and high-risk karyotype; chemotherapy and BSC versus HMA; bone-marrow blast percentage; WBC) reached statistical significance at the two-sided α = 0.05 level. Allo-HSCT is shown despite its wide confidence interval given the small transplant subgroup (*n* = 13) and should be interpreted as exploratory

In the multivariable Fine–Gray model (Table 3, Fig. 2), treatment and karyotype were the dominant independent predictors. BSC (SHR 4.77, 95% CI 1.86–12.22, *P* = 0.001) and chemotherapy (SHR 2.16, 1.04–4.48, *P* = 0.039) carried significantly higher transformation risk than HMA; allo-HSCT was non-significantly protective (SHR 0.52, *P* = 0.261). Intermediate- and high-risk karyotype conferred comparable adverse effects (SHR 3.09 and 2.98, *P* = 0.020 and 0.005). Bone-marrow blast percentage (SHR 1.11 per 1%, *P* = 0.026) and WBC (SHR 1.08 per 10 × 10⁹/L, *P* = 0.005) remained independent; WHO classification did not (*P* = 0.918), consistent with collinearity with blast percentage.

**Table 3:** Multivariate Fine-Gray analysis for AML transformation

Variable	SHR	95% CI	*P* value
WHO (CMML-2 vs. CMML-1)	1.05	0.45–2.44	0.918
Karyotype risk (vs. Low)			
Intermediate	3.09	1.19–8.01	0.020
High	2.98	1.40–6.36	0.005
Treatment (vs. HMA)			
Chemotherapy	2.16	1.04–4.48	0.039
BSC	4.77	1.86–12.22	0.001
Allo-HSCT	0.52	0.17–1.63	0.261
BM blast (per %)	1.11	1.01–1.21	0.026
WBC (per 10 × 10⁹/L)	1.08	1.02–1.14	0.005

WHO classification and allo-HSCT were retained as pre-specified a priori covariates irrespective of *P*-value, as detailed in the Statistical Analysis section. All other variables were selected by backward elimination (*P* < 0.10 threshold)

### Molecular predictors in the NGS-tested subgroup

NGS was performed in 62/102 patients (60.8%). Tested and untested patients did not differ in baseline features or transformation outcomes (12-month CIF 46.2% vs. 44.7%, Gray’s *P* = 0.26; Table S2), and adding NGS-testing status to the multivariable model showed no independent association and did not shift other coefficients (SHR 0.95, *P* = 0.87; Table S3). Within tested patients (Table S4), the most prevalent mutations—ASXL1 (37.1%), TET2 (25.8%), CBL (19.4%), SRSF2 (17.7%)—were not independently predictive. SETBP1 (14.5%; SHR 3.09, 1.32–7.23, *P* = 0.009) and TP53 (9.7%; SHR 3.66, 1.37–9.77, *P* = 0.009) carried substantial prognostic weight, with 7/9 and 5/6 mutated patients transforming.

### Fine–Gray versus Cox estimates

Effect directions and magnitudes were generally concordant between models, with Fine–Gray SHRs modestly attenuated relative to Cox HRs (Table S5). The greatest divergence was allo-HSCT—significantly protective by Cox (HR 0.31, *P* = 0.026) but only a trend by Fine–Gray (SHR 0.45, *P* = 0.143)—reflecting that the Cox model censors competing deaths and thereby overstates the isolated anti-transformation effect of a treatment that also lowers mortality. A smaller analogous attenuation occurred for BSC (Cox HR 2.91 vs. Fine–Gray SHR 2.80).

### Cumulative incidence of transformation

**Fig. 3:**
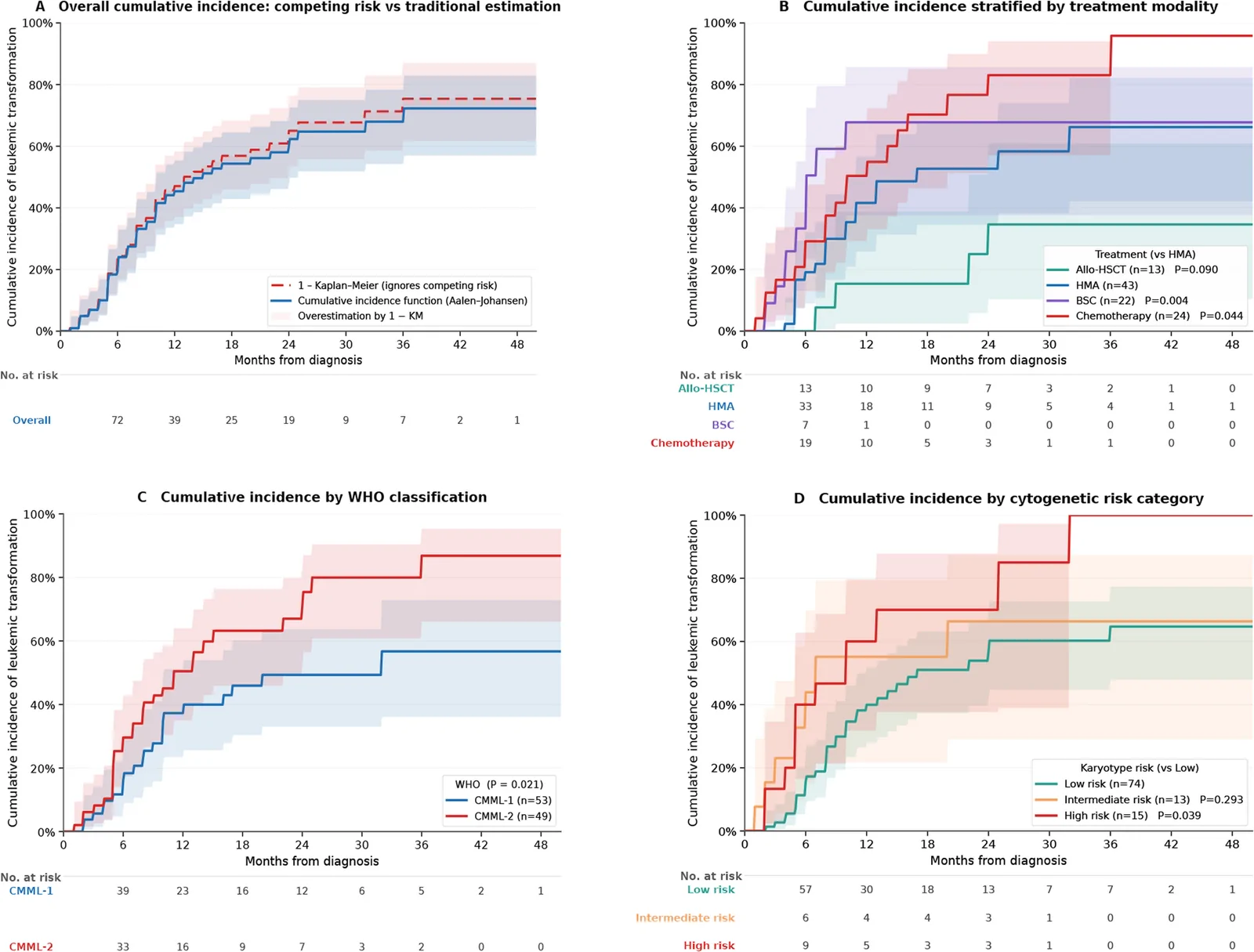
**A **Cumulative incidence of leukemic transformation: competing risk versus traditional estimation. The cumulative incidence function (CIF, solid blue line), which accounts for death as a competing risk, is compared to the traditional 1-Kaplan-Meier estimate (1-KM, dashed red line), which treats competing events as censored. The shaded area highlights the increasing overestimation by the 1-KM method over time. **B **Cumulative incidence of leukemic transformation stratified by treatment modality. Cumulative incidence functions are shown for allo-HSCT (green, *n* = 13), HMA (blue, *n* = 43), BSC (purple, *n* = 22), and chemotherapy (red, *n* = 24). *P* values from Gray’s test comparing each treatment group to HMA as reference are displayed. The allo-HSCT group demonstrated the lowest transformation probability throughout follow-up, while chemotherapy showed the steepest trajectory, exceeding 90% by 36 months. Number at risk for each group is shown below. **C **Cumulative incidence of leukemic transformation by WHO classification. Cumulative incidence functions for CMML-1 (blue, *n* = 53) and CMML-2 (red, *n* = 49) are displayed. CMML-2 patients demonstrated consistently higher transformation probability, reaching 85.7% at 36 months compared to 62.9% for CMML-1. The difference did not achieve statistical significance (Gray’s test *P* = 0.164). Number at risk for each WHO category is shown below. **D **Cumulative incidence of leukemic transformation by cytogenetic risk category. Cumulative incidence functions are stratified by cytogenetic risk: low (green, *n* = 74), intermediate (orange, *n* = 13), and high (red, *n* = 15) according to CPSS criteria. High-risk cytogenetics demonstrated the highest transformation probability, reaching 100% by 36 months. P values from Gray’s test comparing intermediate and high-risk groups to the low-risk reference are shown. Number at risk for each risk category is displayed below

For the full cohort, CIF was 28.1%, 46.9%, 63.4%, and 73.8% at 6, 12, 24, and 36 months (Fig. 3A; Table 4). The 1 − KM estimator systematically overestimated transformation incidence by 0.8% (6 months) to 5.3% (48 months) absolute (Table S6). By treatment (Fig. 3B), allo-HSCT had the lowest cumulative incidence throughout (12- and 24-month CIF 16.7% and 41.7%), HMA intermediate and plateauing after 24 months (12- and 36-month 45.0% and 56.2%), and chemotherapy the steepest sustained trajectory (24- and 36-month 86.2% and 95.0%); BSC showed rapid early transformation with subsequent plateau (6- and 12-month 52.5% and 61.8%). Gray’s test versus HMA was significant for BSC (*P* = 0.029) but not for chemotherapy or allo-HSCT (*P* = 0.134 and 0.220). CMML-2 trended toward higher CIF than CMML-1 (36-month 85.7% vs. 62.9%; Gray’s *P* = 0.164; Fig. 3C). High-risk karyotype showed the highest CIF (36-month 100%) versus low-risk (64.1%; *P* = 0.094 vs. low; Fig. 3D), while FAB subtypes overlapped (Supplementary Fig. 1).

**Table 4:** Cumulative incidence of AML transformation (%) at different time points

Group	*N*	Events	6-mo	12-mo	24-mo	36-mo
Overall	102	54	28.1	46.9	63.4	73.8
By Treatment						
Chemotherapy	24	19	29.2	56.0	86.2	95.0
HMA	43	21	26.6	45.0	52.0	56.2
BSC	22	10	52.5	61.8	61.8	61.8
Allo-HSCT	13	4	8.3	16.7	41.7	66.7
By WHO						
CMML-1	53	22	24.1	43.4	50.7	62.9
CMML-2	49	32	33.0	51.1	74.6	85.7
By Karyotype						
Low	74	38	25.4	42.7	62.4	64.1
Intermediate	13	8	27.0	55.1	70.1	70.1
High	15	8	46.7	60.0	70.0	70.0

CIF estimated using Aalen-Johansen method accounting for competing risk of death

## Discussion

This study represents the first systematic application of competing risk methodology to leukemic transformation in CMML. Four principal findings emerge: (1) traditional Kaplan–Meier analysis overestimates the cumulative incidence of transformation by 0.8–5.3% absolute over 4 years; (2) treatment, cytogenetic risk, bone-marrow blast percentage and WBC are independent predictors after multivariable Fine–Gray adjustment, although the treatment-related estimates should be regarded as exploratory given confounding by indication, baseline imbalance, and immortal-time effects intrinsic to baseline classification; (3) within the NGS-tested subgroup, SETBP1 and TP53 mutations identify small but very high-risk subsets; and (4) the protective association of allo-HSCT seen in cause-specific Cox analysis loses significance under competing-risk modelling, illustrating how the two approaches can diverge for treatments that also reduce mortality.

The systematic overestimation of transformation risk by 1 − KM, although modest in absolute terms, has tangible consequences. For individual prognostication, inflated estimates may prompt unnecessarily aggressive intervention. For trial design, a phase II study powered (80%, α = 0.05) to detect a 30% relative reduction from a KM-based control rate of 67% would require ~ 96 patients per arm, but if the true competing-risk-adjusted control rate is 63%, ~ 110 patients per arm are needed for the same power—a 14% underestimate. Conversely, a non-inferiority trial built against an inflated control rate tolerates a wider margin than the biology justifies. The bias was modest in our cohort (only 7 competing deaths) but would amplify in older, frailer, or longer-followed populations, supporting routine reporting of cumulative incidence functions alongside KM whenever competing mortality is non-negligible [16]. Published transformation rates derived from KM methodology should be interpreted with this bias in mind, particularly in series with longer follow-up or older populations [13].

Our treatment comparisons provide hypothesis-generating signals rather than causal estimates. The 4.77-fold transformation hazard for BSC versus HMA is consistent with a benefit of active disease-modifying therapy when feasible, extending prior data based on response and survival endpoints [6–8]. The higher hazard for chemotherapy than HMA (SHR 2.16) likely reflects selection of patients with more aggressive disease and the modest durability of chemotherapy-induced responses in a disease characterised by persistent clonal haematopoiesis [22]. Residual confounding by comorbidity, ECOG performance status and frailty—none uniformly captured—cannot be excluded for any of these contrasts. The allo-HSCT estimates illustrate a more conceptual issue: by censoring competing deaths, the Cox model attributes their removal from the risk set entirely to treatment effect, conflating anti-transformation efficacy with mortality reduction. The Fine–Gray model retains these patients in the risk set until either event occurs and therefore yields a “real-world” probability of transformation that accounts for the possibility of dying first. Because the subdistribution hazard for allo-HSCT was not significant (SHR 0.52, *P* = 0.261) and the transplant subgroup was small (*n* = 13), our data do not establish an independent anti-transformation effect; if such a benefit exists, its absolute magnitude is likely smaller than the cause-specific Cox estimate implies [23].

Cytogenetic risk emerged as the dominant disease-related predictor: intermediate- and high-risk karyotypes conferred comparable ~ 3-fold hazards relative to low-risk, supporting a dichotomous (normal/favourable versus abnormal) operationalisation and aligning with established CMML prognostic systems [24]. Molecular profiling in the NGS-tested subgroup (62/102, 60.8%; mutations detected in 60) refined this picture without compromising it. Testing was not selective for baseline features or transformation outcomes (12-month CIF 46.2% vs. 44.7%, Gray’s *P* = 0.26), and an NGS-testing indicator added to the multivariable Fine–Gray model showed no independent association (SHR 0.95, *P* = 0.87), arguing against selection bias. Among tested patients, the most prevalent mutations—*ASXL1* (37.1%), *TET2* (25.8%), *CBL* (19.4%), *SRSF2* (17.7%)—did not independently predict transformation in subdistribution analysis, in contrast with their established adverse effect on overall survival in larger series [25, 26]; this divergence likely reflects both limited subgroup power and the conceptual difference between cause-specific survival endpoints and the marginal transformation probability captured by Fine–Gray. Two lower-frequency lesions identified subgroups with very high transformation risk: *SETBP1* (14.5%; SHR 3.09, *P* = 0.009) and *TP53* (9.7%; SHR 3.66, *P* = 0.009), with 7/9 and 5/6 mutated patients transforming, respectively. *SETBP1* mutations mark clonal evolution and aggressive disease in CMML [27], and *TP53* is an established adverse genomic finding across myeloid neoplasms [28]; detection of either should prompt consideration of accelerated transplant evaluation. The independent prognostic significance of continuous bone-marrow blasts (SHR 1.11 per 1%) and WBC (SHR 1.08 per 10 × 10⁹/L) further reinforces that disease burden contributes risk continuously rather than at threshold cut-offs, favouring prognostic models that retain these variables on a continuous scale.

The cumulative incidence of transformation observed here (52.9% overall; 36-month CIF 73.8%) is higher than the 14–30% commonly cited in earlier CMML series [1–3] and warrants comment. Several features of the present cohort are likely contributory rather than indicative of a definitional artefact. CMML-2 accounted for 48.0% of patients and MP-CMML for 58.8%, both proportions exceeding those in many historical series; the median bone marrow blast percentage at presentation was 10.0%, near the upper end of the diagnostic range; and single-centre referral patterns in a tertiary haematology service may have further enriched the cohort for higher-risk disease. Notably, the high transformation rate was observed despite a relatively short median follow-up (12 months), implying that the patients who transformed did so early in their disease course—a pattern more consistent with a biologically enriched, higher-risk cohort than with extended observation time. Transformation was defined uniformly using current WHO criteria and morphologically confirmed in every case, so a definitional artefact is unlikely.

Overall and progression-free survival should remain the principal endpoints in CMML therapeutic studies, as they capture treatment benefit comprehensively and align with patient priorities. Fine–Gray models and CIFs are complementary, answering a more specific question—the marginal probability of a particular event in the face of realistic competing mortality—and should be reported alongside KM whenever cumulative-incidence statements are made. A useful methodological illustration is the allo-HSCT comparison, where the cause-specific Cox model suggested protection but the Fine–Gray subdistribution hazard was not significant (SHR 0.52, *P* = 0.261); although this contrast rests on few competing events (*n* = 7) and a small transplant subgroup (*n* = 13) and is therefore not in itself conclusive, it shows how competing-risk methodology can change the interpretation of an individual covariate even when the overall absolute bias between methods appears small.

Several limitations require emphasis. First, this is a retrospective single-centre analysis with non-random treatment assignment; confounding by indication is intrinsic and cannot be removed by multivariable adjustment alone, so all treatment-effect estimates are exploratory. Second, because CMML is rare, the cohort was assembled by enrolling all consecutive eligible patients during an 11.9-year window rather than from a prospective sample-size calculation; although the overall event count is adequate (54 transformations, 7 competing deaths) and the cohort size is consistent with other single-institution CMML series (typically 60–220 patients [29, 30]), the multivariable Fine–Gray model has an events-per-variable ratio of 6.0, below the conventional ≥ 10 rule of thumb although within the range supported by recent simulation work for testing pre-specified covariates [31], and the allo-HSCT (*n* = 13) and BSC (*n* = 22) subgroups carry correspondingly wide confidence intervals. Third, baseline imbalance was substantial—allo-HSCT recipients were younger and BSC recipients had lower albumin—and residual confounding by comorbidity, performance status and donor availability is likely. Fourth, NGS was performed in 60.8% of patients (mutations detected in 60 of 62 tested), so the molecular findings apply to a subgroup; the sensitivity analysis suggests this did not bias principal estimates, but mutations of lower prevalence than *TP53* could not be reliably examined and contemporary molecularly-integrated scores (CPSS-Mol, Mayo Molecular Model) could not be applied. Fifth, treatment was classified by primary modality at baseline, so patients who initiated 7 + 3 with transplant intent but did not proceed (chemo-refractoriness, toxicity, post-induction unfitness) remained in the chemotherapy arm—an immortal-time and selection bias that inflates chemotherapy-associated hazards and that cannot be resolved within a baseline-classification framework. A time-varying multistate model with chemotherapy and HMA as bridging states and allo-HSCT as an absorbing transition would in principle address this but is not adequately supported by our event counts. Finally, generalisability requires confirmation given single-centre referral patterns.

## Conclusion

Competing risk analysis modifies estimates of leukemic transformation in CMML compared with traditional KM and cause-specific Cox approaches, reducing inflated cumulative incidence and qualitatively altering the interpretation of treatments that also affect mortality, most strikingly allo-HSCT. Adverse cytogenetics is a robust independent predictor of transformation, and within the NGS-tested subgroup *SETBP1* and *TP53* mutations identify subsets at very high transformation risk. Observed treatment differences should be regarded as exploratory given non-random allocation and small subgroup sizes. We recommend that competing risk analysis be reported alongside OS and PFS whenever cumulative-incidence statements are made in CMML and analogous myeloid malignancies; treatment-specific recommendations require validation in larger, ideally multi-centre or prospective cohorts.

## Supplementary Information

Supplementary Material 1.

## Data Availability

The data that support the findings of this study are available from the corresponding author on reasonable request.

## Abbreviations

CMML: Chronic myelomonocytic leukemia
MD-CMML: Myelodysplastic chronic myelomonocytic leukemia
MP-CMML: Myeloproliferative chronic myelomonocytic leukemia
CIF: Cumulative incidence functions
HMA: Hypomethylating agents
BSC: Best supportive care
SHR: Subdistribution hazard
WHO: World Health Organization
allo-HSCT: Allogeneic haematopoietic stem-cell transplantation
KM: Kaplan–Meier
CIF: Cumulative incidence functions
NGS: Next-generation sequencing
CPSS: CMML-specific Prognostic Scoring System
FAB: French- American- British
BM: Bone marrow
WBC: White blood cell count
LDH: Lactate dehydrogenase
