# Supplementary material for: Cumulative incidence and prognostic factors for leukemic transformation in chronic myelomonocytic leukemia: a competing risk analysis
Source: BMC Cancer. 2026 Jun 17;26:1024. doi: 10.1186/s12885-026-16346-y (PMC13508553; doi:10.1186/s12885-026-16346-y)
Supplement: Supplementary file 1 [file 12885_2026_16346_MOESM1_ESM.docx]

**Supplementary Figure 1. Cumulative incidence of leukemic transformation by FAB classification.** Cumulative incidence functions for MD-CMML (blue, n=42) and MP-CMML (red, n=60) are shown. The curves were largely overlapping throughout follow-up, with no significant difference between FAB subtypes, suggesting limited discriminatory power for transformation risk compared to WHO classification and cytogenetic risk.

# Supplementary Tables

**Table S1.** Test of the proportional hazards assumption for covariates included in the final multivariable model.

| **Variable** | **χ² statistic** | **df** | **p-value** |
| --- | --- | --- | --- |
| WHO classification | 0.34 | 1 | 0.56 |
| Karyotype risk (intermediate) | 0.24 | 1 | 0.63 |
| Karyotype risk (high) | 1.91 | 1 | 0.17 |
| Treatment: chemotherapy | 0.05 | 1 | 0.82 |
| Treatment: BSC | 0.01 | 1 | 0.92 |
| Treatment: allo-HSCT | 0.13 | 1 | 0.72 |
| BM blast percentage | 0.83 | 1 | 0.36 |
| WBC count | 0.01 | 1 | 0.93 |
| Global test | 3.42 | 8 | 0.91 |

Proportional hazards were tested using scaled Schoenfeld residuals from the cause-specific Cox model, with the time transformation g(t) = 1 − Kaplan–Meier(t), following the approach of Grambsch and Therneau.¹ For each covariate, the chi-square statistic tests the null hypothesis of a zero slope of scaled Schoenfeld residuals against g(t)—a non-zero slope would indicate a time-dependent effect. The global test is the joint chi-square statistic from all covariates. Reference categories were CMML-1 for WHO classification, low-risk karyotype, and hypomethylating agents (HMA) for treatment. Bone-marrow blast percentage and white blood cell count were modelled as continuous variables. P values >0.05 indicate that the proportional hazards assumption is not violated. No covariate showed a significant departure from proportionality (all individual P >0.10, global χ² = 3.42, df = 8, P = 0.91), supporting the use of time-fixed coefficients in the multivariable analysis.

Abbreviations: allo-HSCT, allogeneic haematopoietic stem-cell transplantation; BM, bone marrow; BSC, best supportive care; df, degrees of freedom; HMA, hypomethylating agent(s); WBC, white blood cell count; WHO, World Health Organization.

**Table S2. Baseline characteristics by NGS testing status (n=102).**

| **Characteristic** | **NGS performed (n=62)** | **NGS not performed (n=40)** | **P value** |
| --- | --- | --- | --- |
| Age, years — mean | 63.5 | 62.7 | 0.75 |
| Female sex, n (%) | 48 (77.4%) | 27 (67.5%) | 0.38 |
| WHO CMML-2, n (%) | 28 (45.2%) | 21 (52.5%) | 0.60 |
| FAB MP-CMML, n (%) | 35 (56.5%) | 25 (62.5%) | 0.69 |
| Bone-marrow blast %, mean | 9.1 | 9.4 | 0.81 |
| High-risk karyotype, n (%) | 9 (14.5%) | 6 (15.0%) | 0.17 (overall) |
| CPSS Intermediate-2 / High, n (%) | 29 (46.8%) | 21 (52.5%) | 0.93 |
| HMA as first-line, n (%) | 27 (43.5%) | 16 (40.0%) | 0.16 (overall) |
| allo-HSCT as first-line, n (%) | 9 (14.5%) | 4 (10.0%) | — |
| **AML transformation, n (%)** | **33 (53.2%)** | **21 (52.5%)** | **1.00** |
| Death (any cause), n (%) | 27 (43.5%) | 14 (35.0%) | 0.51 |
| 12-month CIF for transformation | 46.2% | 44.7% | 0.26 (Gray) |
| 24-month CIF for transformation | 63.2% | 61.1% | — |

*Continuous variables compared by t-test or Mann–Whitney U; categorical variables by chi-square or Fisher exact test. CIF, cumulative incidence function (Aalen–Johansen). Gray’s test by permutation (2 000 replicates) on the integrated squared difference of CIFs.*

**Table S3. Multivariable Fine-Gray model in the full cohort (n=102), with NGS-testing status added as a sensitivity covariate.**

| **Variable** | **SHR** | **95% CI** | **P value** |
| --- | --- | --- | --- |
| WHO CMML-2 vs CMML-1 | 1.27 | 0.55–2.93 | 0.58 |
| Karyotype: high vs low | 3.00 | 1.43–6.30 | 0.004 |
| Karyotype: intermediate vs low | 3.61 | 1.43–9.14 | 0.007 |
| Bone-marrow blast (per %) | 1.08 | 0.99–1.18 | 0.08 |
| Chemotherapy vs HMA | 1.94 | 0.98–3.82 | 0.057 |
| BSC vs HMA | 2.21 | 0.96–5.08 | 0.063 |
| allo-HSCT vs HMA | 0.38 | 0.12–1.16 | 0.089 |
| **NGS performed (yes vs no) — sensitivity covariate** | **0.95** | **0.50–1.79** | **0.87** |

*SHR, subdistribution hazard ratio (Fine-Gray model). The indicator for NGS-testing status is not associated with transformation and does not materially shift the other coefficients.*

**Table S4. Mutation prevalence and association with leukemic transformation (NGS-tested subgroup, n=62).**

| **Gene** | **Mutated, n (%)** | **Transformed / mutated, n** | **SHR** | **95% CI** | **P value** |
| --- | --- | --- | --- | --- | --- |
| ASXL1 | 23 (37.1%) | 12 / 23 | 0.93 | 0.46–1.90 | 0.85 |
| TET2 | 16 (25.8%) | 6 / 16 | 0.58 | 0.24–1.41 | 0.23 |
| CBL | 12 (19.4%) | 6 / 12 | 1.30 | 0.54–3.17 | 0.56 |
| SRSF2 | 11 (17.7%) | 4 / 11 | 0.62 | 0.22–1.76 | 0.37 |
| NRAS | 10 (16.1%) | 5 / 10 | 0.68 | 0.26–1.77 | 0.43 |
| KRAS | 9 (14.5%) | 4 / 9 | 0.61 | 0.21–1.73 | 0.35 |
| **SETBP1** | **9 (14.5%)** | **7 / 9** | **3.09** | **1.32–7.23** | **0.009** |
| RUNX1 | 7 (11.3%) | 3 / 7 | 0.99 | 0.30–3.25 | 0.99 |
| **TP53** | **6 (9.7%)** | **5 / 6** | **3.66** | **1.37–9.77** | **0.009** |
| EZH2 | 6 (9.7%) | 3 / 6 | 0.67 | 0.20–2.19 | 0.50 |

## Table S5. Comparison of Traditional Cox Model vs Competing Risk (Fine-Gray) Model.

| **Variable** | **Cox Model** | | | **Fine-Gray Model** | | |
| --- | --- | --- | --- | --- | --- | --- |
|  | **HR** | **95% CI** | **P** | **SHR** | **95% CI** | **P** |
| Gender (Male vs Female) | 1.16 | 0.66-2.05 | 0.605 | 1.16 | 0.66-2.05 | 0.605 |
| WHO (CMML-2 vs CMML-1) | 1.79 | 1.04-3.08 | 0.036 | 1.79 | 1.04-3.08 | 0.036 |
| Karyotype (Int vs Low) | 1.29 | 0.58-2.85 | 0.537 | 1.46 | 0.65-3.31 | 0.360 |
| Karyotype (High vs Low) | 1.77 | 0.93-3.36 | 0.084 | 1.87 | 0.96-3.61 | 0.064 |
| Treatment (Chemo vs HMA) | 1.72 | 0.98-3.02 | 0.058 | 1.80 | 0.96-3.37 | 0.067 |
| Treatment (BSC vs HMA) | 2.91 | 1.39-6.09 | 0.005 | 2.80 | 1.24-6.31 | 0.013 |
| Treatment (HSCT vs HMA) | 0.31 | 0.11-0.87 | 0.026 | 0.45 | 0.15-1.31 | 0.143 |
| Age (per year) | 0.99 | 0.97-1.01 | 0.432 | 0.99 | 0.97-1.01 | 0.432 |
| BM blast (per %) | 1.10 | 1.04-1.16 | 0.001 | 1.10 | 1.04-1.16 | 0.001 |
| WBC (per 10×10⁹/L) | 1.06 | 1.01-1.11 | 0.022 | 1.06 | 1.01-1.11 | 0.022 |
| Hemoglobin (per 10 g/L) | 0.88 | 0.79-0.97 | 0.014 | 0.88 | 0.79-0.97 | 0.014 |
| LDH (per 100 U/L) | 1.08 | 1.00-1.16 | 0.041 | 1.08 | 1.00-1.16 | 0.041 |
| Albumin (per g/L) | 0.97 | 0.92-1.01 | 0.133 | 0.97 | 0.92-1.01 | 0.133 |

*HR: Cause-specific Hazard Ratio; SHR: Subdistribution Hazard Ratio; Cox model treats competing deaths as censored; Fine-Gray model accounts for competing risks.*

## Table S6. Comparison of CIF vs Traditional 1-KM Estimates.

| **Time** | **CIF (%)** | **1-KM (%)** | **Difference (%)** | **Relative overestimation (%)** |
| --- | --- | --- | --- | --- |
| 6 months | 28.1 | 28.9 | +0.8 | +2.8 |
| 12 months | 46.9 | 49.2 | +2.3 | +4.9 |
| 24 months | 63.4 | 67.2 | +3.8 | +6.0 |
| 36 months | 73.8 | 78.4 | +4.6 | +6.2 |
| 48 months | 79.2 | 84.5 | +5.3 | +6.7 |

*1-KM method overestimates cumulative incidence by ignoring competing risks.*
